# Long-term proton pump inhibitors use and its association with premalignant gastric lesions: a systematic review and meta-analysis

**DOI:** 10.3389/fphar.2023.1244400

**Published:** 2023-08-25

**Authors:** Zeyi Zheng, Ziyu Lu, Yani Song

**Affiliations:** ^1^ School of Traditional Chinese Medicine, Inner Mongolia Medical University, Hohhot, Inner Mongolia, China; ^2^ School of Basic Medicine, Inner Mongolia Medical University, Hohhot, Inner Mongolia, China; ^3^ School of Water Resources and Hydropower Engineering, Wuhan University, Wuhan, Hubei, China

**Keywords:** proton pump inhibitors, gastric cancer, meta-analysis, gastric mucosal atrophy, intestinal metaplasia, enterochromaffin-like cell, gastric polyps

## Abstract

**Background:** Long-term maintenance therapy with proton pump inhibitors (PPIs) is a common treatment strategy for acid-related gastrointestinal diseases. However, concerns have been raised about the potential increased risk of gastric cancer and related precancerous lesions with long-term PPI use. This systematic review and meta-analysis aimed to evaluate this potential risk.

**Methods:** We searched PubMed, Embase, and the Cochrane Central Register of Controlled Trials for randomised controlled trials published before 1 March 2023, with no language restrictions. The primary endpoint was the occurrence and progression of gastric mucosal atrophy, intestinal metaplasia, Enterochromaffin-like (ECL) cell hyperplasia, gastric polyps, and gastric cancer during the trial and follow-up. Data were analysed using a random effects model.

**Results:** Of the 4,868 identified studies, 10 met the inclusion criteria and were included in our analysis, comprising 27,283 participants. Compared with other treatments, PPI maintenance therapy for more than 6 months was associated with an increased risk of ECL cell hyperplasia (OR 3.01; 95% CI 1.29 to 7.04; *p* = 0.01). However, no significant increase was found in the risk of gastric mucosal atrophy (OR 1.01; 95% CI 0.55 to 1.85; *p* = 0.97), intestinal metaplasia (OR 1.14; 95% CI 0.49 to 2.68; *p* = 0.76), gastric polyps (OR 1.13; 95% CI 0.68 to 1.89; *p* = 0.64), or gastric cancer (OR 1.06; 95% CI 0.79 to 1.43; *p* = 0.71).

**Conclusion:** This systematic review and meta-analysis does not support an increased risk of gastric cancer or related precancerous lesions with long-term PPI maintenance therapy. However, long-term PPI use should be monitored for potential complications such as ECL cell hyperplasia. Further studies are needed to confirm these findings and evaluate the safety of PPI maintenance therapy for acid-related gastrointestinal diseases.

**Systematic Review Registration:** https://www.crd.york.ac.uk/prospero/, Identifier: PROSPERO (CRD42022379692).

## 1 Introduction

Gastric cancer is among the top five causes of death globally (Colquhoun et al., 2015), with a 5-year survival rate of 25%–30% (Allemani et al., 2015). The primary reason for this unfavourable prognosis is the late-stage diagnosis of most gastric cancer patients. Therefore, reducing gastric cancer mortality requires significant attention to managing its pathogenic factors and early signs.

The progression from normal gastric mucosa to early intestinal gastric cancer is typically a sequence of “normal gastric mucosa → chronic gastritis → atrophy → intestinal metaplasia → dysplasia → early gastric cancer” (Correa et al., 1975; Correa, 1992). Atrophy, intestinal metaplasia, and dysplasia are broadly referred to as gastric precancerous lesions due to their potential malignant risk (Piazuelo et al., 2021). Gastric polyps are limited epithelial protrusions of the epithelial mucosa, with fundus glandular polyps (FGPs) and hyperplastic polyps (HPs) being the most common pathological types (Ren et al., 2018). Adenomatous polyps have a high malignant potential and are often associated with gastric adenocarcinoma (Park and Lauwers, 2008). Molecular studies have shown that gastric polyps, excluding adenomatous polyps, also exhibit molecular changes that may lead to tumour progression and may have unknown risks of malignant transformation (Carmack et al., 2009).

Proton pump inhibitors (PPIs), widely used globally for treating acid-related diseases such as gastroesophageal reflux disease (Dilaghi et al., 2022) and peptic ulcer (Malfertheiner et al., 2017), are often used as maintenance therapy to prevent symptom recurrence. Long-term PPI use often results in increased circulating gastrin levels, which can affect gastrointestinal tissue. However, sustained high levels of circulating gastrin can lead to hyperplasia of gastric parietal cells and enterochromaffin-like cells in the gastric mucosa (Lundell et al., 2015), potentially inducing the development of fundic gland polyps (Kim, 2021) and other histopathological changes that may increase the risk of gastric cancer. (Hagiwara et al., 2011). The safety and adverse effects of long-term PPI use are becoming a concern.

The impact of long-term PPI use on gastric cancer-related lesions has been extensively studied, but the results have been inconsistent, leading to controversy about the safety of PPI maintenance therapy (Yadlapati and Kahrilas, 2018). Therefore, a meta-analysis is needed to comprehensively evaluate the association between long-term PPI use and gastric cancer and its related lesions. We conducted a systematic review and meta-analysis of existing randomized controlled trials to assess the adverse effects of long-term PPI use on gastric cancer, gastric mucosal atrophy, intestinal metaplasia, ECL cell hyperplasia, and gastric polyps to elucidate and improve the safety of PPI maintenance therapy.

Previous meta-analyses have shown varying results. Song’s meta-analysis found no clear evidence of an increased risk of gastric mucosal atrophy and intestinal metaplasia but a more significant association with ECL cell hyperplasia (Song et al., 2014). Tran-Duy (Tran-Duy et al., 2016) and Martin (Martin et al., 2016)’s showed an association with an increased risk of fundic gland polyps and possibly an increased risk of gastric cancer. Three recent meta-analyses by Segna (Segna et al., 2021), Guo (Guo et al., 2023) and Peng (Peng et al., 2023) showed that PPI use was significantly associated with gastric cancer, but no duration-dependent effects of PPI with gastric cancer risk were found at < 1 year, 1–3 years, and more than 3 years. This meta-analysis includes more recent high-quality studies related to PPI maintenance therapy and reduced heterogeneity, allowing for a more comprehensive evaluation of the adverse effects on gastric cancer-related lesions.

## 2 Materials and methods

### 2.1 Search strategy

We conducted a literature search in PUBMED, EMBASE, and the Cochrane Central Register of Controlled Trials electronic databases to identify all published and unpublished randomised controlled trials up to 1 March 2023. An additional manual search of conference abstracts was performed to ascertain experimental details. Bibliographies of relevant studies were also manually searched. Two investigators independently conducted searches using the keywords “Stomach Neoplasms” and “proton pump inhibitor.” The specific search strategy is detailed in the attached table.

### 2.2 Selection criteria

The inclusion criteria encompassed randomised controlled trials (RCTs) examining the long-term use of PPIs in adult patients. The study necessitated one group of participants on maintenance therapy with PPIs and a control group using other treatment strategies for an intervention period exceeding 6 months. Participants in both groups underwent upper gastrointestinal endoscopy at the start and conclusion of the trial. They reported the number or proportion of participants with gastric mucosal lesions such as gastric cancer, atrophy, intestinal metaplasia, ECL cell hyperplasia, and gastric polyps. The primary outcome was the study’s ability to provide sufficient data to estimate the odds ratio (OR) between the occurrence and progression of each gastric cancer-related lesion and the use of PPI.

Articles without original data, studies that did not report data related to “gastric cancer, atrophy, intestinal metaplasia, ECL cell hyperplasia, and gastric polyps,” and studies with duplicate reports were excluded.

Two independent investigators screened titles and abstracts to include all potential studies in the search results. They independently screened them in full text to identify eligible studies and document reasons for exclusion. In cases of uncertainty regarding study inclusion, the two review authors discussed to reach a resolution, and if necessary, a third expert was consulted.

**FIGURE 1:**
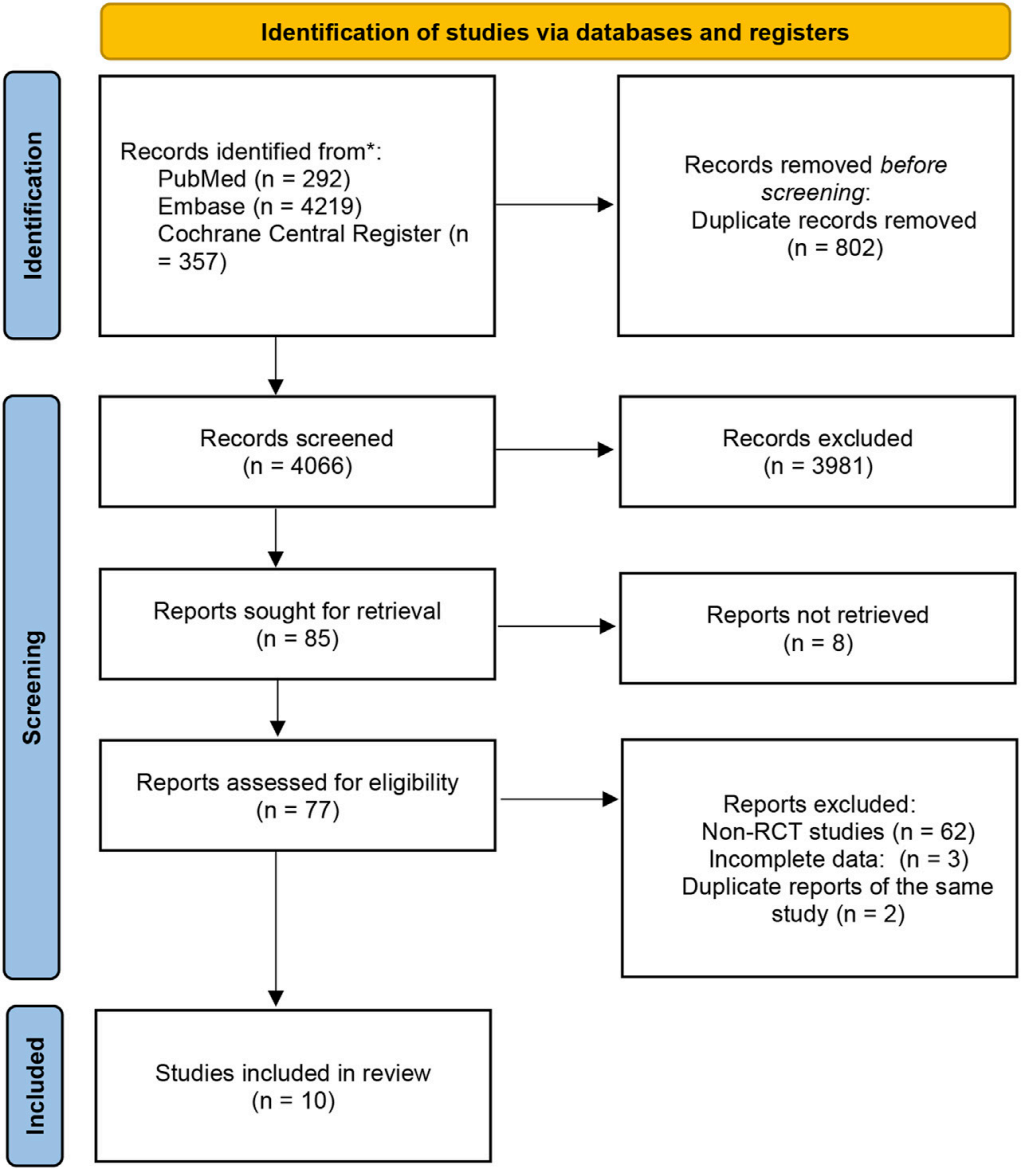
PRISMA flowchart illustrating the process of screening and selection of studies.

This section was conducted in accordance with the Preferred Reporting Items for Systematic Reviews and Meta-Analyses (PRISMA) flowchart (Page et al., 2021), and registered at the International Prospective Register of Systematic Reviews (number CRD42022379692) (Figure 1. PRISMA flowchart illustrating the process of screening and selection of studies).

### 2.3 Data extraction and quality assessment

Two independent investigators extracted data from each study using a pre-designed data extraction form. Extracted data included the first author’s surname, year of publication, type of experiment, number of participants, age, sex, baseline diagnosis, inclusion and exclusion criteria, treatment and experimental interventions, duration of intervention, primary outcome, and number of events. For unpublished studies, we referred to their practical design article (Uemura et al., 2018) and extracted data from their most recent conference presentation (Kushima et al., 2022). Two independent investigators assessed the quality of each study using the Cochrane risk of bias tool (Higgins et al., 2011), with any discrepancies resolved by a third investigator.

### 2.4 Statistical analysis

For dichotomous variables, we assumed that patients who discontinued were negative and that data reporting only the change in the number of events and the possibility of repeated reporting were positive. We used Stata16 software to generate meta-analysis and forest plots. Binary data were analysed into odds ratio (OR) with a 95% confidence interval (CI) using the DerSimonian-Laird random effects model to evaluate the overall sensitivity and specificity of the model, thereby fully accounting for the additional uncertainty related to inter-study differences in the effects of different interventions. We calculated the parameters Q, I^2^, I^2^ (≥50%), and P (<0.05) (Higgins and Thompson, 2002; Higgins et al., 2003) to assess heterogeneity of values. If feasible, we planned to conduct a subgroup analysis to evaluate the potential impact of the incident location, *Helicobacter pylori* infection, PPI dose, and exposure time. In cases of high heterogeneity in the main results, we would conduct a sensitivity analysis to assess the impact of individual studies on aggregate statistics, by excluding one study at a time from the meta-analysis. If the number of included studies was sufficient (≥ 10), we would explore potential publication bias by constructing a funnel plot of the effect size of each trial relative to the standard error, and evaluate the asymmetry of the funnel plot using Begg and Egger tests, defining significant publication bias as a *p*-value <0.1.

## 3 Result

Initially, we identified a total of 4,868 studies from electronic databases. Out of these, 802 were duplicates. After screening by title and abstract, we selected 85 studies for further evaluation. However, we could not retrieve 8 reports. Out of the remaining 77 studies, 62 were non-randomized controlled trials (RCTs) or review articles. Additionally, we found 2 reports to be duplicates of the same study, while 3 studies were excluded due to incomplete data. Among the remaining nine studies, one of them (Genta et al., 2003) reported adverse reaction results of two studies (Johnson et al., 2001; Vakil et al., 2001) with identical designs. We found the original texts of these two studies for quality analysis. One study (Haruma et al., 2021) is a meeting abstract for a study that is still in progress. We retrieved the study design (Uemura et al., 2018) for this study and the 4-year study data presented at the meeting in May 2022 (Kushima et al., 2022).

**FIGURE 2:**
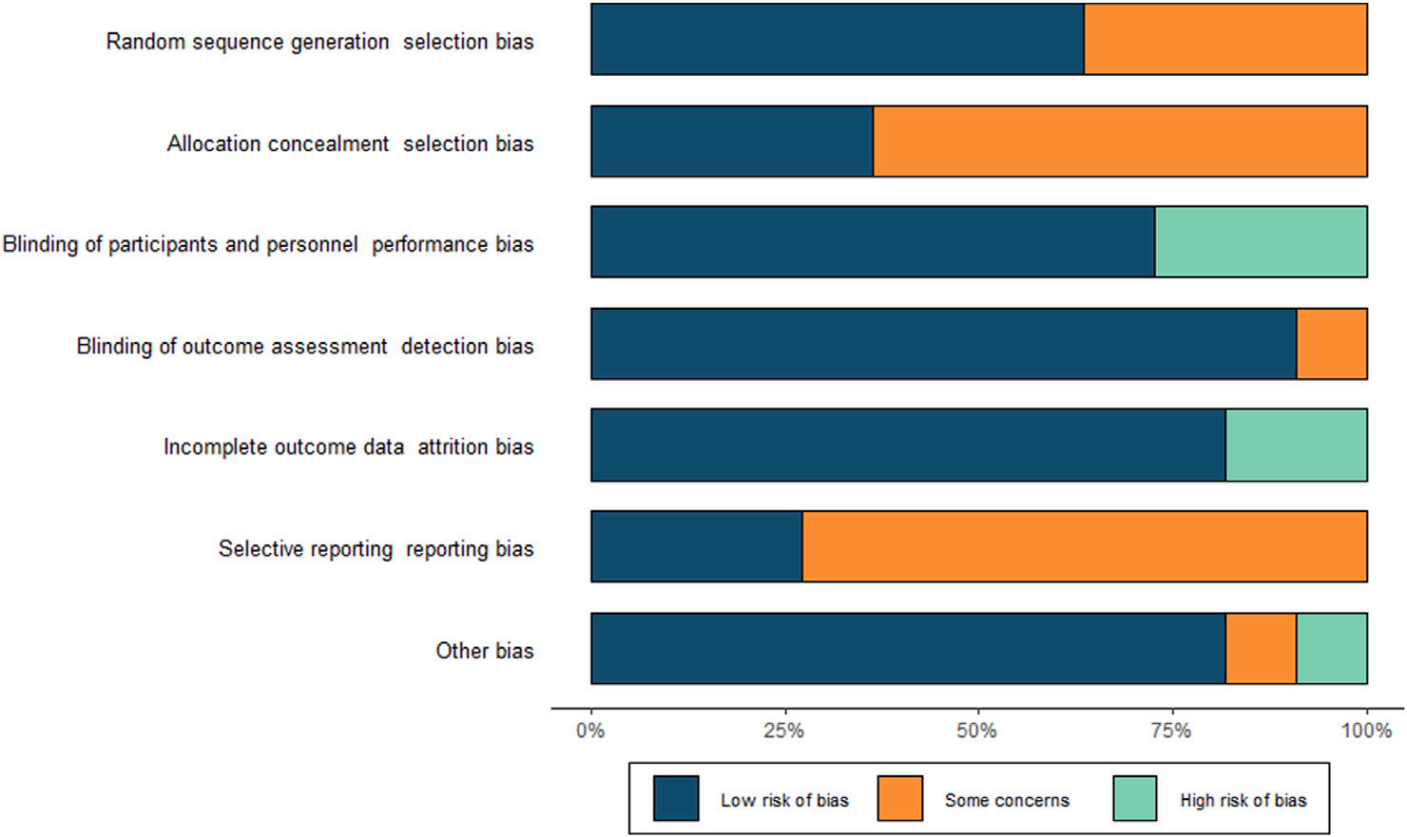
Risk of bias graph.

**FIGURE 3:**
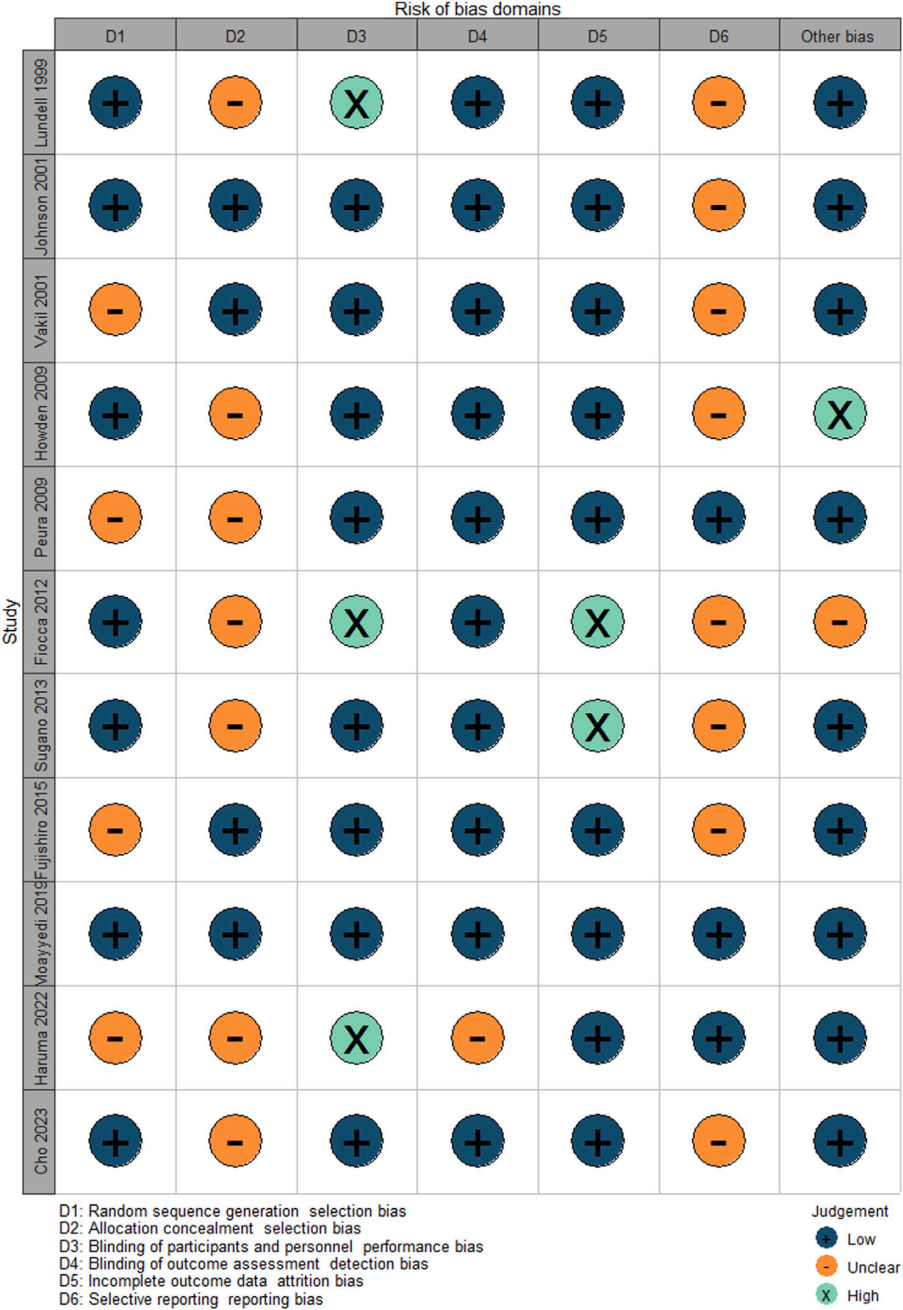
Risk of bias summary.

We included ten randomised controlled studies for the effect of proton pump inhibitors on gastric cancer-related lesions, comprising 27,283 subjects (Table 1. Summary of study characteristics) (Figure 2. Risk of bias Figure 3. Risk of bias summary.).

**TABLE 1:** Summary of study characteristics.

Study	Year	Treatment in both study groups	Duration of interventions	PPI users/PPI none users	Outcome	Events in PPI users/PPI none users	Age	Male	Countries/Regions
Lundell et al.	1999	omeprazole (20–40 mg once daily) vs. antireflux surgery	up to 3 years	155/143	Worsening atrophic gastritis scores	10/11	18–77	OME 75% ARS 76%	Nordic countries
					Worsening intestinal metaplasia scores	2/3			
					ECL cell hyperplasia (diffuse/liner/micronodular)	7/2			
Genta et al.	2003	esomeprazole (10,20,40 mg once daily) vs. placebo	up to 6 months	519/169	Changes From Baseline in Gastritis atrophy at Antral and Corporal Biopsy Sites	11/1	18–75	no mentioned	United States
					Changes From Baseline in intestinal metaplasia at Antral and Corporal Biopsy Sites	7/4			
					ECL Cell Scores at Baseline and Final Biopsies	27/1			
Howden et al.	2009	Dexlansoprazole (60,90 mg once daily) vs. placebo	up to 6 months	311/140	Improvement of intestinal metaplasia scores	10/5	≥18	MR90: 82 MR60: 83 Placebo:70	United States
					Prevalence of enterochromaffin-like cell hyperplasia or adenocarcinoma	0/0			
Peura et al.	2009	Dexlansoprazole (30,60,90 mg qd) vs. lansoprazole (30 mg qd) vs. placebo	276.2 ± 134.67 days	5633/896	The number of patients with gastric polyps	19/1	18–90	DLAS 46% LAS 53% Placebo 34%	United States
					The number of patients with intestinal metaplasia	5/0			
					The number of patients with enterochromaffin-like cell hyperplasia	0/0			
Fiocca et al.	2012	Esomeprazole (20 mg once daily) vs. laparoscopic surgery	up to 5 years	158/180	The number of atrophy at each time point (antrum/corpus)	4/6	18–70	LARS 72% ESO 82%	Europe
					The number of intestinal metaplasia at each time point (antrum/corpus)	11/12			
					The histogram of ECL cell hyperplasia at each time point	14/3			
Sugano et al.	2013	Esomeprazole (20 mg daily) vs. Placebo.	up to 72 weeks	214/213	The number of participants with gastric polyps at summary of adverse events	2/1	≥20	ESO 80.8% Placebo 79.1%	Japan
		Low-dose acetylsalicylic acid (81–324 mg/day for ≤5 of 7 days each week) and Gefarnate (50 mg twice daily)							Korea
									Taiwan
Fujishiro et al.	2015	Rabeprazole (10 mg daily) vs. Rabeprazole (5 mg daily)	up to 76 weeks	204/201	Number of participants with Gastric cancer	4/1	35–90	10 mg 74.5% 5 mg 76.1%	Japan
Moayyedi et al.	2019	Pantoprazole (40 mg once daily) vs. placebo	up to 3 years	8,791/8,807	The number of patients with atrophy	19/26	mean 67.6 ± 8.1	PAT 78% Placebo 79%	America
					The number of patients with gastric cancer	86/83			Europe
									Asia Pacific and other
Haruma et al.	2022	Lansoprazole (15 or 30 mg once daily) vs. Vonoprazan (10 or 20 mg once daily)	up to 5 years	67/135	The number of patients with ECL cell hyperplasia	1/2	≥ 20	LPZ 61% VPZ 72%	Japan
Cho et al.	2023	lansoprazole 15 mg or tegoprazan 25 mg once daily	up to 24 weeks	174/173	The number of participants with gastric polyps at summary of adverse events	3/4	20–75	LPZ 110 TPZ 114	Korean

Gastric mucosal atrophy and intestinal metaplasia: Four studies evaluated gastric mucosal atrophy. One study reported the changes in the number of participants with gastric mucosal atrophy and intestinal metaplasia at the endpoint compared to the baseline for each degree of progression (Lundell et al., 1999). Another study reported only the endpoint number of occurrences of gastric mucosal atrophy (Moayyedi et al., 2019). A pooled set (Genta et al., 2003) of adverse effects of two studies (Johnson et al., 2001; Vakil et al., 2001) reported the number of participants whose gastric mucosal atrophy and intestinal metaplasia aggravated and decreased in the antrum and gastric corpus, respectively, compared to the baseline. One study also reported atrophy and intestinal metaplasia at each follow-up time point by dividing the gastric antrum and gastric corpus (Fiocca et al., 2012). However, we could not obtain trends from this study, and we had no way of knowing whether the participants with events in the antrum and gastric corpus overlapped. We first assume that the experimental group using PPI has the most events and the control group has the least events as a premise for including the data in this study. To avoid bias, we subsequently excluded this study and conducted an analysis again to observe whether the direction of the meta-analysis results after exclusion is consistent with that under this extreme condition.

**FIGURE 4:**
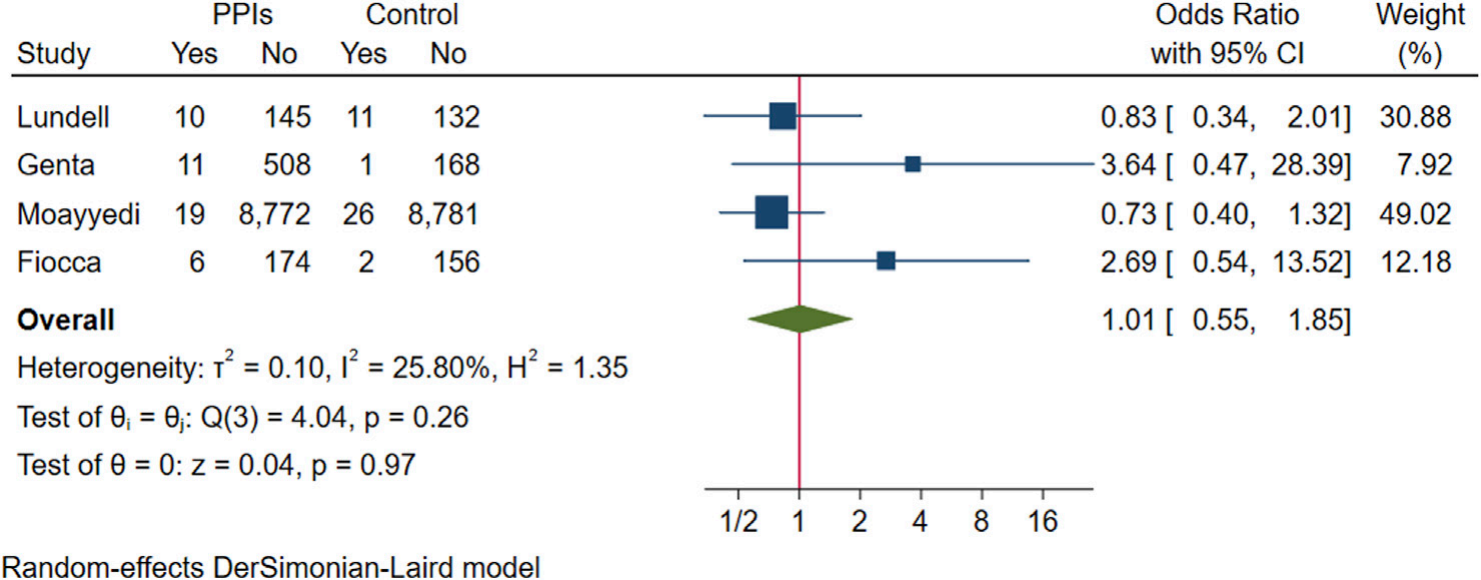
Forest plots of odds ratios for atrophy in participants receiving proton pump inhibitors compared with subjects not receiving proton pump inhibitors.

In a pooled analysis of the above four studies, progressive worsening of gastric mucosal atrophy was found in 46 of 9,645 participants treated with PPI maintenance for more than 6 months and in 40 of 9,277 participants in the control group. No statistical difference was found between the groups (OR 1.01; 95% CI 0.55 to 1.85; *p*-value = 0.97), indicating that most participants showed improvement or no change in the degree of gastric mucosal atrophy (Figure 4. Forest plots of odds ratios for atrophy in participants receiving proton pump inhibitors compared with subjects not receiving proton pump inhibitors.). When re-analysed after excluding studies assuming extreme cases of Fiocca (Fiocca et al., 2012), the results of the meta-analysis were in the same direction (OR 0.84; 95% CI 0.5 to 1.41; *p*-value = 0.51) and the I^2^ decreased from 25.8% to 7.48%.

**FIGURE 5:**
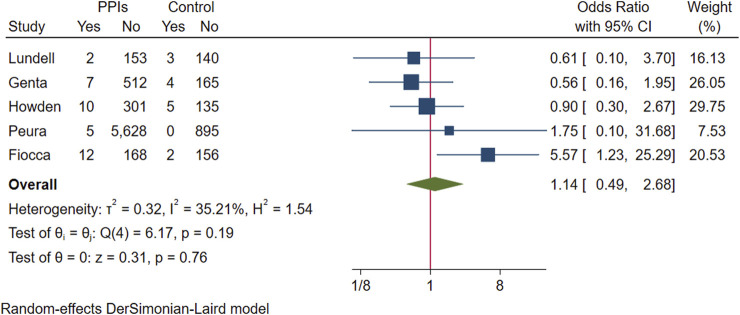
Forest plots of odds ratios for intestinal metaplasia in participants receiving proton pump inhibitors compared with subjects not receiving proton pump inhibitors.

We pooled five RCTs evaluating 8,303 participants, in which one study reported the incidence of intestinal metaplasia in participants with endpoint gastric biopsies (Howden et al., 2009), and another study reported the number of participants who had intestinal metaplasia in gastric biopsies (Peura et al., 2009). Intestinal metaplasia was present on endpoint gastric biopsies in 36 participants in the PPI group and 14 in the control group, with no evidence that intestinal metaplasia was associated with exacerbation and long-term use of PPIs (OR 1.14; 95% CI 0.49 to 2.68; *p*-value = 0.76) (Figure 5. Forest plots of odds ratios for intestinal metaplasia in participants receiving proton pump inhibitors compared with subjects not receiving proton pump inhibitors.). Likewise, after excluding studies that assumed extreme cases of Fiocca and conducting a subsequent analysis, the meta-analysis results were consistent in direction (OR 0.75; 95% CI 0.36 to 1.55; *p*-value = 0.44), and I^2^ decreased from 35.21% to 0.

Enterochromaffin-like cell hyperplasia: Six studies evaluated changes in ECL cells. Two studies reported no occurrences of ECL cell hyperplasia during the study period (Howden et al., 2009; Peura et al., 2009). One study reported changes in each level of ECL cell abnormalities in participants from baseline to endpoint (Lundell et al., 1999). Another study provided a histogram representation of the number of patients with ECL cell hyperplasia, which we manually entered for measurement (Fiocca et al., 2012). Additionally, one study reported the number of participants with ECL cell hyperplasia on gastric tissue biopsy at the endpoint and the proportion of these participants with participants whose ECL cell scores worsened (Genta et al., 2003). We also retrieved data from one ongoing study with a 5-year duration (Kushima et al., 2022) which presented its fourth-year gastric biopsy data at a conference in May 2022.

**FIGURE 6:**
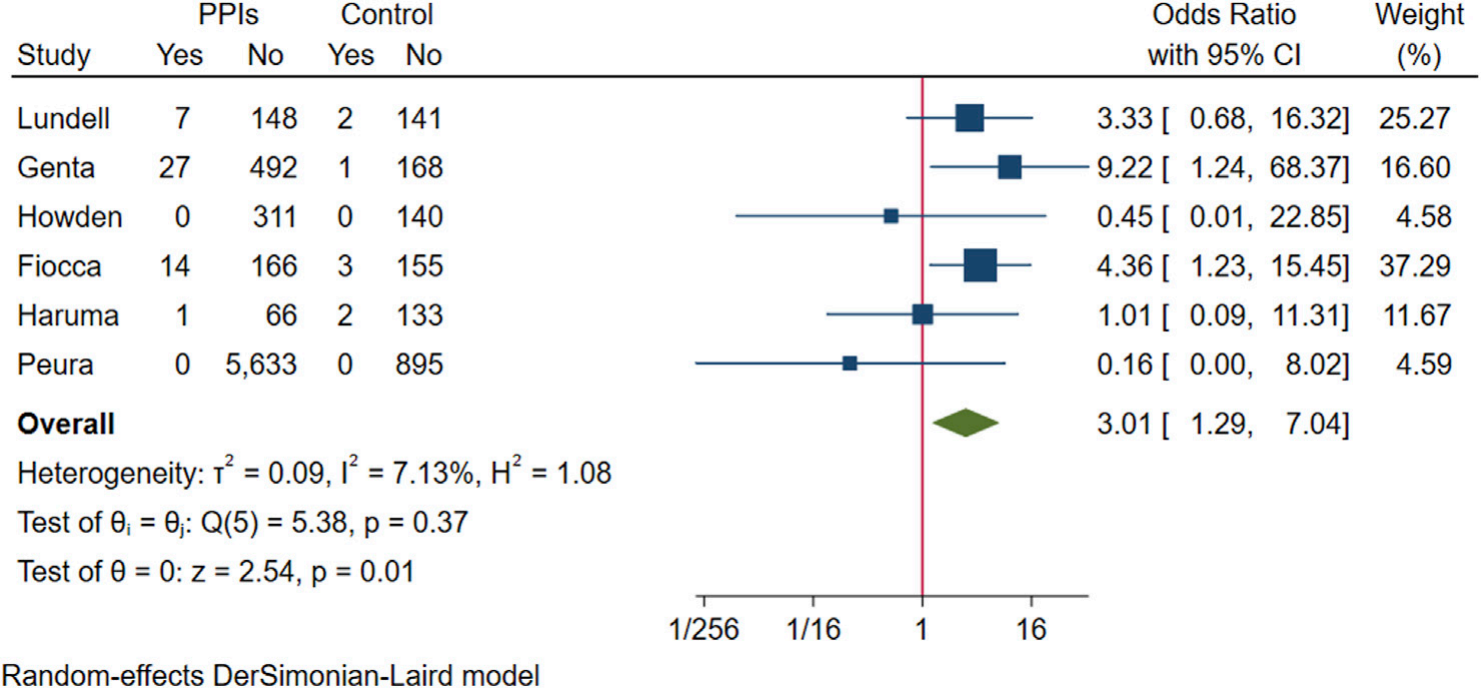
Forest plots of odds ratios for ECL cell hyperplastic in participants receiving proton pump inhibitors compared with subjects not receiving proton pump inhibitors.

**FIGURE 7:**
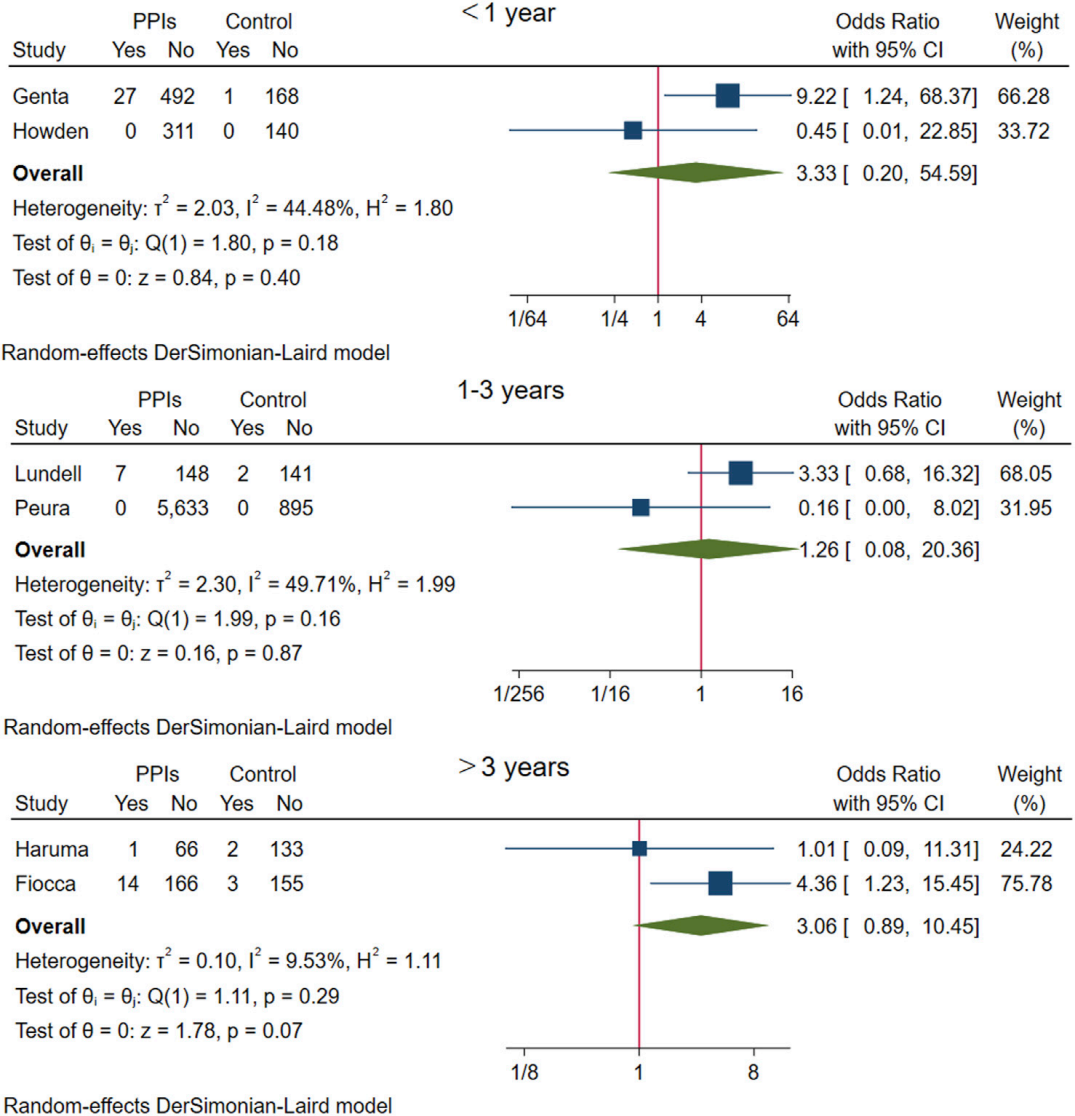
Forest plots of the odds of ECL cell proliferation in participants who received proton pump inhibitors compared to those who did not receive proton pump inhibitors by different observation duration.

**FIGURE 8:**
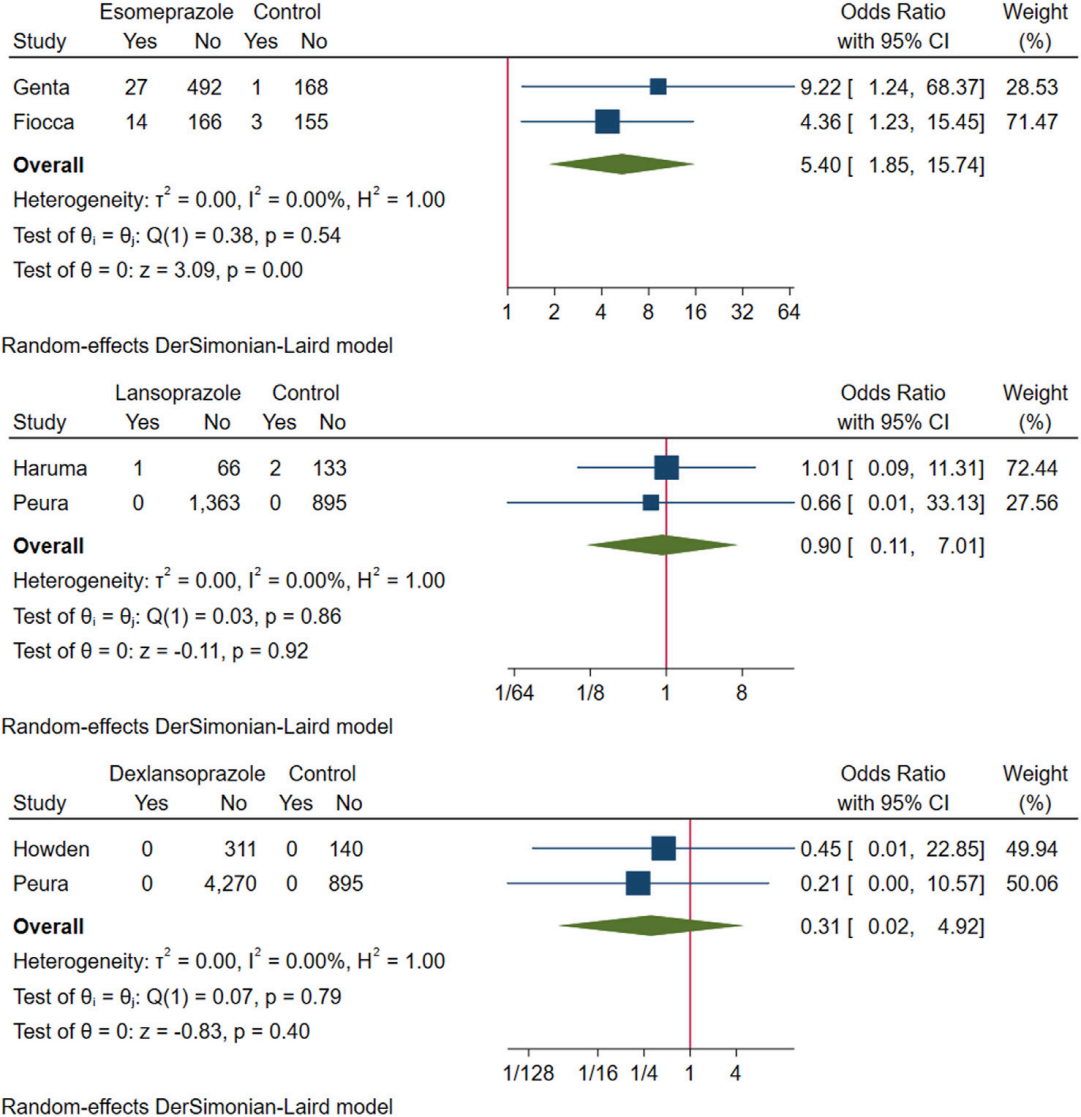
Forest plots of the odds of ECL cell proliferation in participants who received proton pump inhibitors compared to those who did not receive proton pump inhibitors by using different types of PPIs.

The above six studies were pooled and analysed. Among the 6,865 participants who used PPI maintenance therapy for more than 6 months, 49 had deterioration of ECL cell score, and only 8 of the 1,640 participants in the control group had a breakdown of ECL score. This meta-analysis showed that long-term PPI maintenance therapy users had an increased risk of worsening ECL cell scores relative to non-PPI users (OR 3.01; 95% CI 1.29 to 7.04; *p*-value = 0.01) (Figure 6. Forest plots of odds ratios for ECL cell hyperplastic in participants receiving proton pump inhibitors compared with subjects not receiving proton pump inhibitors.). On this basis, we performed a subgroup analysis regarding the duration of observation and the type of PPI, and the results showed that the negative effect of PPI maintenance treatment on ECL cell proliferation did not seem to be dependent on the duration of observation (<1 year: OR 3.33; 95% CI 0.2 to 54.59; *p*-value = 0.4, 1–3 years: OR 1.26; 95% CI 0.08 to 20.36; *p*-value = 0.87, >3 years: OR 3.06; 95% CI 0.89 to 10.45; *p*-value = 0.07), no trend was observed (Figure 7. Forest plots of the odds of ECL cell proliferation in participants who received proton pump inhibitors compared to those who did not receive proton pump inhibitors by different observation duration.), but it seemed to be related to the type of PPI, and the studies with Esomeprazole (OR 5.4; 95% CI 1.85 to 15.74; *p*-value = 0) were more significant than those with Lansoprazole (OR 0.9; 95% CI 0.11 to 7.01; *p*-value = 0.92) or Dexlansoprazole (OR 0.31; 95% CI 0.02 to 4.92; *p*-value = 0.4) (Figure 8. Forest plots of the odds of ECL cell proliferation in participants who received proton pump inhibitors compared to those who did not receive proton pump inhibitors by using different types of PPIs.).

Gastric polyps and Cancers: Four studies evaluated participants for gastric polyps over the course of the study. One study recorded the number of patients with endoscopic fundic gland polyps at baseline and during each follow-up period (Fiocca et al., 2012). Four other studies reported the number of participants who developed gastric polyps during the study as adverse reactions (Peura et al., 2009; Sugano et al., 2014; Kushima et al., 2022; Cho et al., 2023), but the location of the gastric polyp was not indicated. Three studies clearly described participants with gastrointestinal cancers; one research stated that no patients were found to have cancer during the study (Lundell et al., 1999), and another showed the number of participants with cancer in the adverse effect table (Moayyedi et al., 2019). Two groups of patients in one study used 10 mg and 5 mg of rabeprazole once daily (Fujishiro et al., 2015), respectively, which we included as the experimental and control groups were included in the study.

**FIGURE 9:**
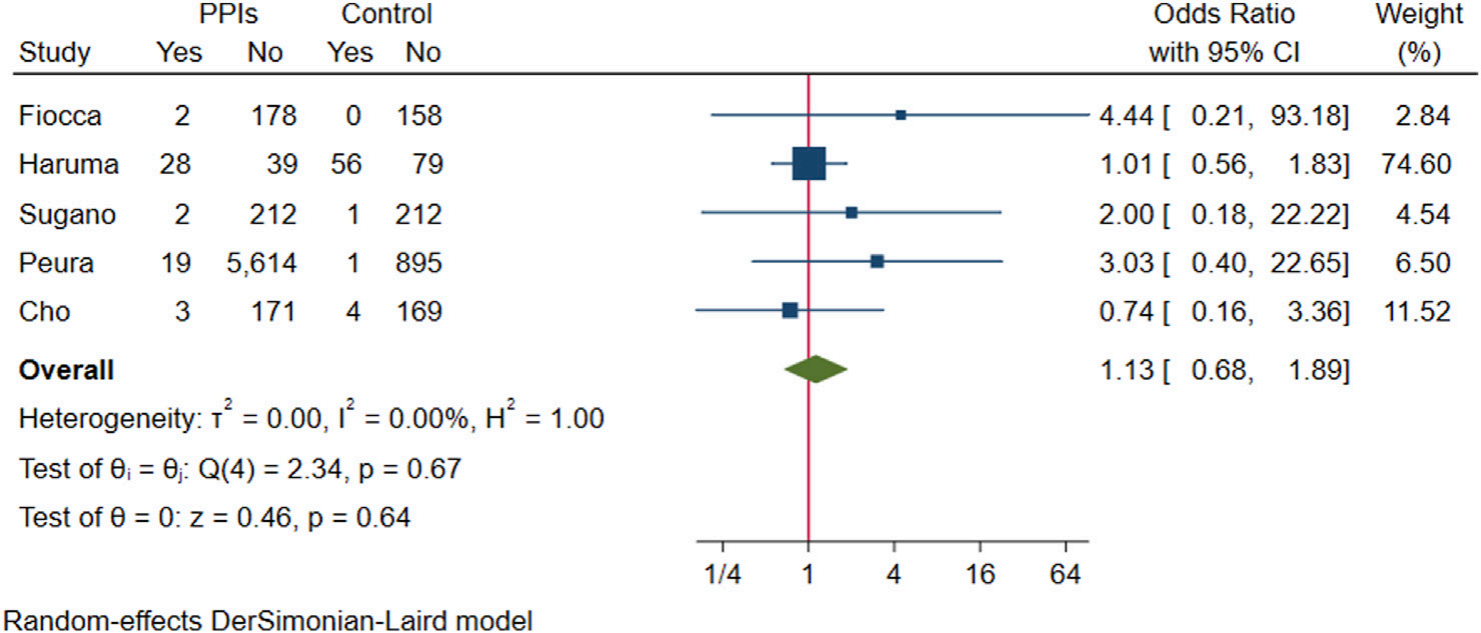
Forest plots of odds ratios for gastric polyps in participants receiving proton pump inhibitors compared with subjects not receiving proton pump inhibitors.

Pooling the above five studies, 55 of 6,268 participants who received PPI maintenance therapy for more than 6 months developed gastric polyps, gastric polyps were detected in 62 of the 1,575 participants in the control group, and there was no evidence that the presence of gastric polyps was associated with long-term PPI use (OR 1.13; 95% CI 0.68 to 1.89; *p*-value = 0.64) (Figure 9. Forest plots of odds ratios for gastric polyps in participants receiving proton pump inhibitors compared with subjects not receiving proton pump inhibitors.).

**FIGURE 10:**
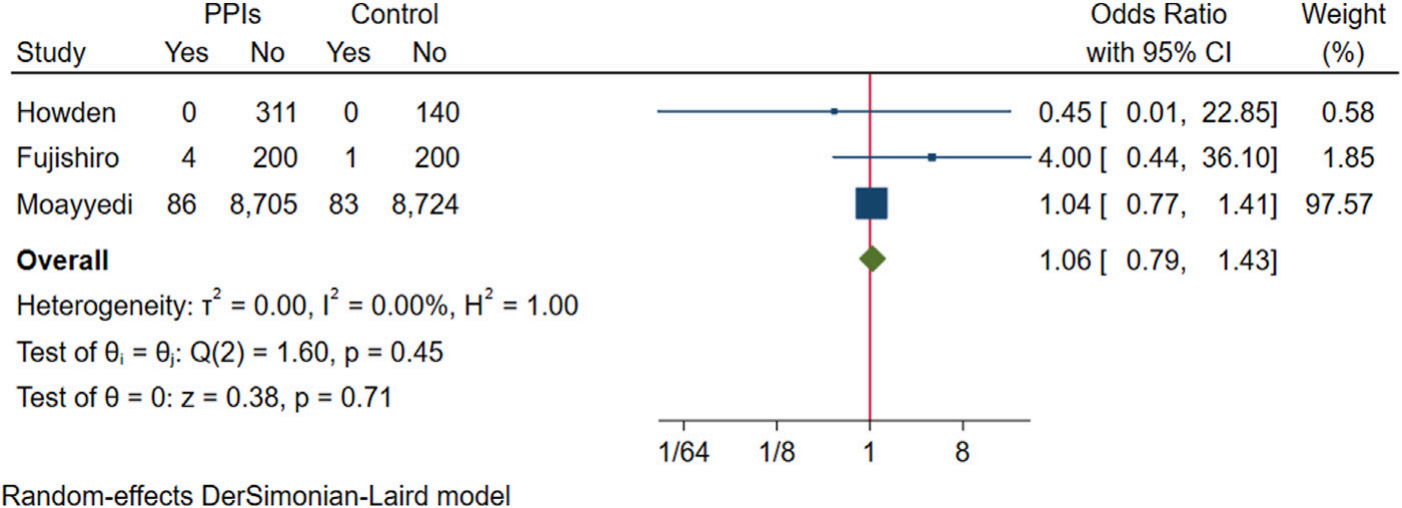
Forest plots of odds ratios for gastric cancer in participants receiving proton pump inhibitors compared with subjects not receiving proton pump inhibitors.

In a pooled analysis of three studies with gastric cancer statistics, 90 of the 9,306 participants in the experimental group developed to gastric cancer, and 84 of the 9,148 participants in the control group were found to have gastric cancer. The difference in prevalence between the two groups was not statistically significant (OR 1.06; 95% CI 0.79 to 1.43; *p*-value = 0.71) (Figure 10. Forest plots of odds ratios for gastric cancer in participants receiving proton pump inhibitors compared with subjects not receiving proton pump inhibitors.).

## 4 Discussion

Our analysis found no evidence that long-term maintenance therapy with PPIs causes or exacerbates gastric mucosal atrophy, intestinal metaplasia, gastric polyps, or gastric cancer in users compared to other treatments. However, we did find a correlation between PPI use and ECL cell proliferation, which may depend on the type of PPI used rather than the duration of use. Our results are consistent with Song’s analysis (Song et al., 2014), but incorporate more recent RCT studies to improve the analysis’s quality and minimise bias. Overall, these findings suggest that long-term maintenance therapy with PPIs can be safely used as a therapeutic strategy in clinical practice. However, the potential risk of ECL cell proliferation associated with high gastrin levels over a long time must be considered.

In recent years, concerns have arisen that long-term use of PPIs may increase the risk of gastric cancer-related disease (Salvo et al., 2021), and various hypotheses have been proposed regarding its causes and mechanisms. One hypothesis is that prolonged acid suppression therapy may alter the stomach’s acid environment, allowing colonisation by various bacteria (Yang and Metz, 2010), including *H. pylori*, which can damage the gastric oxidative glands (Parsons et al., 2017) and potentially trigger malignant transformation, possibly accompanied by a targeted transfer of gastric flora due to profound acid suppression (Malfertheiner et al., 2017). However, two studies (Lundell et al., 1999; Fiocca et al., 2012) found no significant increase in gastric atrophy and intestinal metaplasia in H. pylori-positive patients on long-term PPI maintenance therapy, and the difference in lesions in the gastric body and sinus in the two studies (Genta et al., 2003; Fiocca et al., 2012) was not significant. Although we initially planned to perform a subgroup analysis regarding *H. pylori* and lesion site, we were unable to do so due to the limited number of relevant trials. Based on the available studies, it appears that acid suppression alone is unlikely to have adverse effects on the human gastric mucosa. Nonetheless, the dysbiosis of the gastrointestinal flora caused by long-term PPI use (Sanduleanu et al., 2001; Lo and Chan, 2013) and the association of *H. pylori* with gastrointestinal cancers remain areas of concern (Lee et al., 2013; Mera et al., 2018; Yan et al., 2022). Further research is needed to determine the best approach to managing these risks.

Ever since the observation of hypergastrinemia-induced ECL cell hyperplasia, a pre-cancerous condition, in the carcinogenicity study of omeprazole conducted on rats (Havu, 1986), the issue concerning the association between proton pump inhibitors (PPIs) and gastric cancer risk has never ceased. According to another study, when 75% of the acid-producing portion of a rat’s stomach is removed, hypergastrinemia occurs, and over time, there is a proliferation of ECL cells (Mattsson et al., 1991). High-dose, potent, and long-acting acid secretion inhibitors can alter the pH of the gastrointestinal system, affecting acid feedback regulation, leading to an increase in gastrin levels, and subsequently resulting in hypergastrinemia (Håkanson and Sundler, 1990; Veysey-Smith et al., 2021), which has a direct trophic effect on ECL cells (Waldum and Mjønes, 2020). This may be the reason why ECL cell proliferation appears to be related to the long-term use of PPI maintenance therapy. There is no threshold concentration for the trophic effect of gastrin on ECL cells (Peghini et al., 2002), and long-term high gastrin levels will inevitably cause the proliferation of ECL cells (Rais et al., 2022), which may develop into tumour cells, thus increasing the risk of cancer development in the long run, which may be intestinal gastric adenocarcinoma or neuroendocrine cancer (Waldum and Mjønes, 2020; Rais et al., 2022). However, the process of inducing gastric tumours requires a considerable amount of time and may only manifest after several decades. We found that the studies using esomeprazole (Genta et al., 2003; Fiocca et al., 2012) had more ECL cell hyperplasia in participants treated with PPI maintenance therapy compared to those using dexlansoprazole (Howden et al., 2009; Peura et al., 2009) or lansoprazole (Peura et al., 2009; Haruma et al., 2021). In the classification of Proton Pump Inhibitors (PPIs), we categorize them based on their chemical structure and mechanism of action, primarily into benzimidazole and thiazole classes. The benzimidazole class includes omeprazole, lansoprazole, and esomeprazole, while the thiazole class includes pantoprazole and rabeprazole. The majority of PPIs are metabolized primarily through the hepatic cytochrome P450 enzyme system. For instance, omeprazole and lansoprazole are mainly metabolized through CYP2C19 and CYP3A4, while esomeprazole is primarily metabolized through CYP2C19 (Li et al., 2004). There are differences among various PPIs in terms of inhibiting gastric acid secretion, onset time, duration, and protective effects on the gastric mucosa. Generally, the acid-suppressing effect of esomeprazole is considered the strongest (Edwards et al., 2001; Edwards et al., 2006), followed by rabeprazole, then omeprazole, and finally lansoprazole (Miner et al., 2003). This is primarily due to their differing metabolic rates and pathways in the body, which influence their pharmacological effects. Esomeprazole, the S-isomer of omeprazole, has a more stable molecular structure, is more readily absorbed by the human body, and has a longer half-life in gastric acid, enabling it to continuously inhibit gastric acid secretion. Esomeprazole directly acts on the proton pumps of gastric wall cells, inhibiting their production of gastric acid, and its acid-suppressing effect is stronger than other proton pump inhibitors, this may trigger a reflection on the choice of clinical PPI type. The ECL cell density did not increase in many participants, suggesting that not all patients require strong inhibition of gastric acid secretion. Clinically, the appropriate type of PPI and the appropriate dosage should be chosen according to individual needs. The risk of elevated gastrin and consequent ECL cell hyperplasia due to long-term PPI use may lead to gastric neuroendocrine tumours or gastric adenocarcinoma is not negligible (Cavalcoli et al., 2015). However, this may be the result of a combination of causes. The presence of *H. pylori* and the patient’s original gastric precancerous lesions are also considered to be the causes of elevated gastrin (Veysey-Smith et al., 2021). Therefore, in the long-term management of PPI maintenance therapy, it is important to consider the risk of ECL cell proliferation and manage any *H. pylori* infection or gastric precancerous lesions.

Regarding gastric polyps and gastric cancer, we did not restrict the location of polyps to include all RCT studies that reported gastric polyps because of the small number of studies addressing fundic gland polyps. Our results reflect some controversy compared to previous pooled analyses by Tran-Duy (Tran-Duy et al., 2016) and F.C. Martin (Martin et al., 2016) which were based on population cohort studies and mostly cross-sectional studies conducted during a specific period. We also found differing conclusions in this area of the literature (Hong et al., 2011; Krishnan et al., 2012; Brusselaers et al., 2020) when we reviewed RCT studies. In gastric cancer, there have also been several meta-analyses (Segna et al., 2021; Guo et al., 2023; Peng et al., 2023) based on case-control studies and retrospective cohort studies in recent years that support the idea that PPI use elevates the risk of gastric cancer, reaching different results than our analysis. The reasons for this may lie mainly in the very limited number of relevant high-quality RCT studies, previous meta-analyses have relied on case-control studies and retrospective cohort studies, which leads to baseline imbalances triggered by inclusion and exclusion criteria, as well as potential difficulties in dosing control and prolonged follow-up, resulting in high heterogeneity. In contrast, our meta-analysis included only existing RCT studies, resulting in lower heterogeneity and different findings from those of recent meta-analyses. Our study offers a unique contribution to the literature by focusing exclusively on high-quality RCT studies, providing a clearer picture of the relationship between PPI use and gastric polyps and cancer. However, we still need more well-designed, high-quality studies to address the current controversy.

In the analyses of gastric atrophy and intestinal metaplasia, after including the data under extreme assumptions, I^2^ = 25.8% for gastric atrophy and I^2^ = 35.21% for intestinal metaplasia, and after excluding this data the I^2^ decreased to 7.48% and 0, respectively, with the same direction of meta-analysis results before and after exclusion, suggesting that the scale of inclusion of data from this study does not affect the final conclusions of the analysis. In the analysis regarding ECL cells, I^2^ = 7.13%, and I^2^ = 0 in the rest of the analyses. *p* > 0.05 was seen in all Q-tests, and the minimum value in several studies was *p* = 0.19. No significant heterogeneity was observed in our research. This is reassuring. However, there are some limitations in the following aspects. First, the number of pieces of literature included in this study is still relatively limited. We sought as much relevant literature as possible while ensuring the quality of the literature and included trials that had not yet been completed but for which data were published annually; second, since the publication of relevant data differed among the literature, and some literature only published the prevalence rate per year, and we could not identify the trend of change. We did not receive a reply after sending an email to the authors to ask about it. We accumulated this data and included it in the analysis, the lack of response to our email inquiries also resulted in some bias; third, the study had diverse control group intervention modalities, including placebo, surgery, and other therapeutic drugs, the association of the disease with the experimental group might not be stable due to the diverse intervention modalities in the control group; fourth, the primary outcomes of some of the literature did not match the present study. It mainly reported data of interest in the form of secondary results or adverse events found during the primary outcome, and we sought reports of relevant events in the eligible full texts, but there may still be incomplete or non-specific statistics for this part of the data; finally, some of the pieces of the literature may also have factors of bias such as open-label design and pharmaceutical industry funding, and other unidentified biases. This study exclusively incorporated Randomised Controlled Trials (RCTs), without considering real-world cohort studies. This was primarily due to the fact that RCTs are considered the gold standard for assessing intervention effects, given their design which minimizes bias to the greatest extent possible. However, we acknowledge that real-world cohort studies, offering a broader patient population and longer follow-up data, could potentially influence our results. In the literature where cohort studies were included, we found a significant association between long-term use of PPIs and premalignant gastric lesions. This could be attributed to the fact that cohort studies typically encompass a wider patient population, including those who might be excluded in RCTs, such as the elderly and patients with multiple chronic diseases. Furthermore, cohort studies usually have a longer follow-up period, which might allow us to observe the potential impacts of long-term PPI use, a feat quite challenging in RCTs. However, the occurrence of carcinogenesis requires a considerable length of time, possibly spanning decades, hence our results may be biased. Therefore, the conclusion should be that PPI treatment within the follow-up period of the included RCTs, ranging from 6 to 36 weeks, will not induce gastric cancer at the end of treatment, but long-term maintenance treatment with PPIs may increase susceptibility to gastric cancer, making individuals more prone to gastric cancer in their later years. Future research should consider incorporating real-world cohort studies to more comprehensively assess the association between long-term use of PPIs and premalignant gastric lesions.

PPIs are being used more frequently while their application is gradually expanding (Kinoshita et al., 2018), and their relationship with adverse effects such as pneumonia (Zirk-Sadowski et al., 2018) and osteoporosis (Park et al., 2022) has received attention. Despite the extensive use of Proton Pump Inhibitors (PPIs), the influence of long-term maintenance therapy on the risk of developing gastric cancer-related diseases remains uncertain. This necessitates further exploration in high-quality clinical studies, with this as the primary outcome. Nevertheless, our meta-analysis of the existing literature reveals no significant correlation between PPI maintenance therapy and an increased risk of gastric mucosal atrophy, intestinal metaplasia, or gastric polyps within a maximum follow-up period of 3 years. However, it does lead to ECL cell hyperplasia, a consequence of excessive gastrin triggered by strong acid suppression, which heightens the patient’s susceptibility to gastric cancer. Therefore, our findings suggest that PPI therapy can be safely and effectively employed for the treatment of patients with gastrointestinal diseases requiring long-term maintenance therapy. However, caution should be exercised when considering the use of PPIs, and the potential risks and benefits of treatment should be meticulously evaluated for each patient. Not all patients necessitate treatment that strongly suppresses gastric acid secretion, such as the most potent acid-suppressing PPIs or PPIs at effective doses.

## Data Availability

The raw data supporting the conclusions of this article will be made available by the authors, without undue reservation.
