# Supplementary material for: Long-term proton pump inhibitors use and its association with premalignant gastric lesions: a systematic review and meta-analysis
Source: Front Pharmacol. 2023 Aug 25;14:1244400. doi: 10.3389/fphar.2023.1244400 (PMC10492503; doi:10.3389/fphar.2023.1244400)

Supplementary Material

Long-term Proton Pump Inhibitors Use and Its Association with Malignant Gastric Lesions: A Systematic Review and Meta-analysis

Z. Y. Zheng*, Z. Y. Lu, Y. N. Song

*** Correspondence:** Z. Y. Zheng, Inner Mongolia Medical University, Hohhot, Inner Mongolia, China. Email: zzy798343105@outlook.com

# Supplementary Figures and Tables

For more information on Supplementary Material and for details on the different file types accepted, please see [here](https://www.frontiersin.org/guidelines/author-guidelines#supplementary-material).

## Supplementary Tables

| **Supplementary Table 1.** Detailed Search Strategy for the Systematic Review | |
| --- | --- |
| Pubmed | ((Stomach Neoplasms [mh]) OR Stomach Neoplasms OR (Neoplasm, Stomach) OR (Stomach Neoplasm) OR (Neoplasms, Stomach) OR (Gastric Neoplasms) OR (Gastric Neoplasm) OR (Neoplasm, Gastric) OR (Neoplasms, Gastric) OR (Cancer of Stomach) OR (Stomach Cancers) OR (Gastric Cancer) OR (Cancer, Gastric) OR (Cancers, Gastric) OR (Gastric Cancers) OR (Stomach Cancer) OR (Cancer, Stomach) OR (Cancers, Stomach) OR (Cancer of the Stomach) OR (Gastric Cancer, Familial Diffuse) OR (atrophic gastritis) OR (Carcinoma in situ) OR (Carcinoma, Preinvasive) OR (Preinvasive Carcinoma) OR (Carcinoma, Intraepithelial) OR (Intraepithelial Carcinoma) OR (Neoplasms, Intraepithelial) OR (Intraepithelial Neoplasm) OR (Neoplasm, Intraepithelial) OR (Intraepithelial Neoplasms) OR (intestinal metaplasia) AND ((proton pump inhibitor [mh] ) OR proton pump inhibitor OR (Inhibitors, Proton Pump) OR (Proton Pump Inhibitor) OR (Inhibitor, Proton Pump) OR Pump Inhibitor, Proton) AND (randomized controlled trial[Publication Type] OR (randomized[Title/Abstract]) OR (controlled clinical trail[Title/Abstract]) OR trial[Title/Abstract]) |
| Embase | #1. 'stomach tumor'/exp OR 'stomach tumor' OR 'stomach neoplasms' OR 'neoplasm, stomach' OR 'stomach neoplasm' OR 'neoplasms, stomach' OR 'gastric neoplasms' OR 'gastric neoplasm' OR 'neoplasm, gastric' OR 'neoplasms, gastric' OR 'cancer of stomach' OR 'stomach cancers' OR 'gastric cancer' OR 'cancer, gastric' OR 'cancers, gastric' OR 'gastric cancers' OR 'stomach cancer' OR 'cancer, stomach' OR 'cancers, stomach' OR 'cancer of the stomach' OR 'gastric cancer, familial diffuse' OR 'atrophic gastritis' OR 'intestinal metaplasia' OR 'atrophic gastritides' OR 'atrophic gastritis' OR 'gastritides, atrophic' OR 'enterochromaffin like cells' OR 'enterochromaffin-like cell' OR 'ecl cells' OR 'ecl cell' OR 'gastric polyp' #2. 'proton pump inhibitor'/exp OR 'proton pump inhibitor' OR 'inhibitors, proton pump' OR 'proton pump inhibitor' OR 'inhibitor, proton pump' OR 'inhibitor, proton pump' OR 'pump inhibitor, proton' #3. #1 AND #2 #4. 'clinical':ti,ab AND 'trial':ti,ab OR 'clinical trial'/exp OR random* OR 'drug therapy':lnk #5. #4 AND #3 |
| Cochrane Central Register of Controlled Trials | ((proton pump inhibitor OR Inhibitors, Proton Pump OR Proton Pump Inhibitor OR Inhibitor, Proton Pump OR Pump Inhibitor, Proton)) AND (((Stomach Neoplasms OR Neoplasm, Stomach OR Stomach Neoplasm OR Neoplasms, Stomach OR Gastric Neoplasms OR Gastric Neoplasm OR Neoplasm, Gastric OR Neoplasms, Gastric OR Cancer of Stomach OR Stomach Cancers OR Gastric Cancer OR Cancer, Gastric OR Cancers, Gastric OR Gastric Cancers OR Stomach Cancer OR Cancer, Stomach OR Cancers, Stomach OR Cancer of the Stomach OR Gastric Cancer, Familial Diffuse OR atrophic gastritis OR intestinal metaplasia OR Atrophic Gastritides OR Atrophic Gastritis OR Gastritides, Atrophic OR Enterochromaffin like Cells OR Enterochromaffin-like Cell OR ECL Cells OR ECL Cell OR gastric polyp))) Filters: Clinical Trial, Randomized Controlled Trial |

## Supplementary Figures

**Supplementary Figure 1.** Forest plots of odds ratios for atrophy in participants receiving proton pump inhibitors compared with subjects not receiving proton pump inhibitors. (After excluding Fiocca's study)


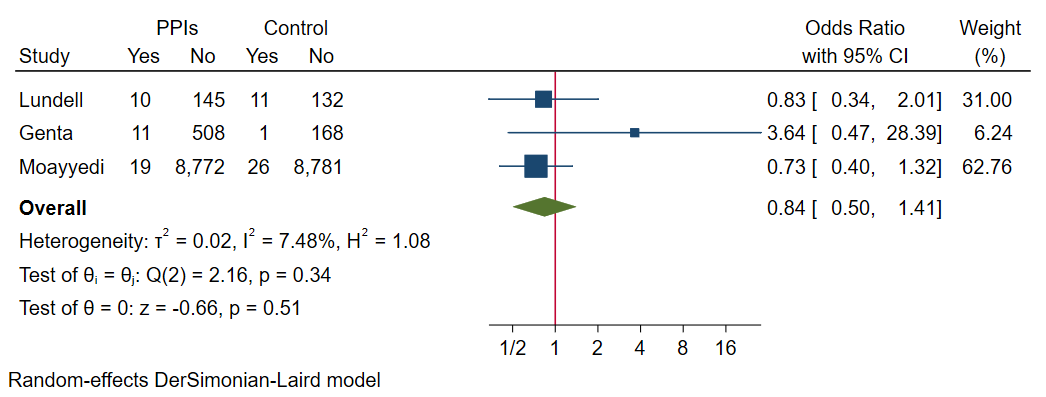


**Supplementary Figure 2.** Forest plots of odds ratios for intestinal metaplasia in participants receiving proton pump inhibitors compared with subjects not receiving proton pump inhibitors. (After excluding Fiocca's study)


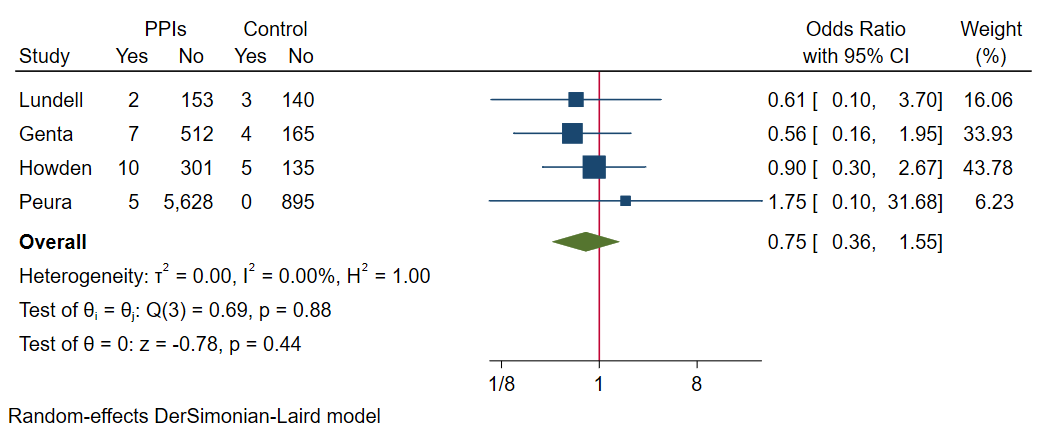

Supplement: Supplementary file 1 [file Table1.DOCX]
