# Supplementary material for: Long-term proton pump inhibitors use and its association with premalignant gastric lesions: a systematic review and meta-analysis
Source: Front Pharmacol. 2023 Aug 25;14:1244400. doi: 10.3389/fphar.2023.1244400 (PMC10492503; doi:10.3389/fphar.2023.1244400)
Supplement: Supplementary file 2 [file DataSheet1.PDF]

PRISMA 2020 Checklist

Section and Topic	Item #	Checklist item	Location where item is reported
**TITLE**			
Title	1	Long-term Proton Pump Inhibitors Use and Its Association with Malignant Gastric Lesions: A Systematic Review and Meta-analysis	1-2
**ABSTRACT**			
Abstract	2	Background: Long-term maintenance therapy with proton pump inhibitors (PPIs) is a common treatment strategy for acid-related gastrointestinal diseases. However, concerns have been raised about the potential increased risk of gastric cancer and related precancerous lesions with long-term PPI use. This systematic review and meta-analysis aimed to evaluate this potential risk.<br>Methods: We searched PubMed, Embase, and the Cochrane Central Register of Controlled Trials for randomised controlled trials published before March 1st, 2023, with no language restrictions. The primary endpoint was the occurrence and progression of gastric mucosal atrophy, intestinal metaplasia, Enterochromaffin-like (ECL) cell hyperplasia, gastric polyps, and gastric cancer during the trial and follow-up. Data were analysed using a random effects model.<br>Results: Of the 4868 identified studies, 10 met the inclusion criteria and were included in our analysis, comprising 27283 participants. Compared with other treatments, PPI maintenance therapy for more than six months was associated with an increased risk of ECL cell hyperplasia (OR 3.01; 95% CI 1.29 to 7.04; P=0.01). However, no significant increase was found in the risk of gastric mucosal atrophy (OR 1.01; 95% CI 0.55 to 1.85; P=0.97), intestinal metaplasia (OR 1.14; 95% CI 0.49 to 2.68; P=0.76), gastric polyps (OR 1.13; 95% CI 0.68 to 1.89; P=0.64), or gastric cancer (OR 1.06; 95% CI 0.79 to 1.43; P=0.71).<br>Conclusions: This systematic review and meta-analysis does not support an increased risk of gastric cancer or related precancerous lesions with long-term PPI maintenance therapy. However, long-term PPI use should be monitored for potential complications such as ECL cell hyperplasia. Further studies are needed to confirm these findings and evaluate the safety of PPI maintenance therapy for acid-related gastrointestinal diseases.	11-37
**INTRODUCTION**			
Rationale	3	Long-term use of PPI is often accompanied by an increase in the circulating gastrin level, which can have effects on gastrointestinal tissue. However, sustained high levels of circulating gastrin can lead to hyperplasia of gastric parietal cells and enterochromaffin-like cells in the gastric mucosa, potentially inducing the development of fundic gland polyps and other histopathological changes that may increase the risk of gastric cancer, as has been observed in rodents. The safety and adverse effects of long-term PPI use are becoming a concern. Whether long-term PPI use has a negative effect on gastric cancer-related lesions has been extensively studied, the results are inconsistent, leading to controversy about the safety of PPI maintenance therapy.	55-63
Objectives	4	We conducted a systematic review and meta-analysis of existing randomised controlled trials to assess the adverse effects of long-term PPI use on gastric cancer, gastric mucosal atrophy, intestinal metaplasia, ECL cell hyperplastic, and gastric polyps to elucidate and improve the safety of PPI maintenance therapy.	64-67
**METHODS**			
Eligibility criteria	5	Population: We included RCTs of adult (age ⩾18 years) patients treated with proton pump inhibitors for more than six months.<br>Intervention: We considered all treatments with proton pump inhibitors for more than six months.<br>Comparator(s): Patients treated with placebo, surgery or other non-proton pump inhibitor drugs.<br>Outcomes: The primary outcome was that the study could provide sufficient data to estimate the odds ratio (OR) between the occurrence and progression of each gastric cancer-related lesion and the use of PPI. Participants in both groups underwent upper gastrointestinal endoscopy at the beginning and end of the trial. They reported the number or proportion of participants with gastric mucosal lesions such as gastric cancer, atrophy, intestinal metaplasia, ECL cell hyperplasia and gastric polyps.<br>We excluded articles without original data, studies that did not report data related to "gastric cancer, atrophy, intestinal metaplasia, ECL cell hyperplasia, and gastric polyps", and studies with duplicate reports.	84-94
Information sources	6	We performed a literature search in PUBMED, EMBASE, and Cochrane Central Register of Controlled Trials electronic databases, to identify all published and unpublished randomised controlled trials before 1st December 2022.	77-79

Section and Topic	Item #	Checklist item	Location where item is reported
Search strategy	7	PubMed:<br>((Stomach Neoplasms [mh]) OR Stomach Neoplasms OR (Neoplasm, Stomach) OR (Stomach Neoplasm) OR (Neoplasms, Stomach) OR (Gastric Neoplasms) OR (Gastric Neoplasm) OR (Neoplasm, Gastric) OR (Neoplasms, Gastric) OR (Cancer of Stomach) OR (Stomach Cancers) OR (Gastric Cancer) OR (Cancer, Gastric) OR (Cancers, Gastric) OR (Gastric Cancers) OR (Stomach Cancer) OR (Cancer, Stomach) OR (Cancers, Stomach) OR (Cancer of the Stomach) OR (Gastric Cancer, Familial Diffuse) OR (atrophic gastritis) OR (Carcinoma in situ) OR (Carcinoma, Preinvasive) OR (Preinvasive Carcinoma) OR (Carcinoma, Intraepithelial) OR (Intraepithelial Carcinoma) OR (Neoplasms, Intraepithelial) OR (Intraepithelial Neoplasm) OR (Neoplasm, Intraepithelial) OR (Intraepithelial Neoplasms) OR (intestinal metaplasia) AND ((proton pump inhibitor [mh]) OR proton pump inhibitor OR (Inhibitors, Proton Pump) OR (Proton Pump Inhibitor) OR (Inhibitor, Proton Pump) OR Pump Inhibitor, Proton) AND (randomized controlled trial[Publication Type] OR (randomized[Title/Abstract]) OR (controlled clinical trail[Title/Abstract]) OR trial[Title/Abstract])<br>Embase:<br>#1. 'stomach tumor'/exp OR 'stomach tumor' OR 'stomach neoplasms' OR 'neoplasm, stomach' OR 'stomach neoplasm' OR 'neoplasms, stomach' OR 'gastric neoplasms' OR 'gastric neoplasm' OR 'neoplasm, gastric' OR 'neoplasms, gastric' OR 'cancer of stomach' OR 'stomach cancers' OR 'gastric cancer' OR 'cancer, gastric' OR 'cancers, gastric' OR 'gastric cancers' OR 'stomach cancer' OR 'cancer, stomach' OR 'cancers, stomach' OR 'cancer of the stomach' OR 'gastric cancer, familial diffuse' OR 'atrophic gastritis' OR 'intestinal metaplasia' OR 'atrophic gastritides' OR 'atrophic gastritis' OR 'gastritides, atrophic' OR 'enterochromaffin like cells' OR 'enterochromaffin-like cell' OR 'ecl cells' OR 'ecl cell' OR 'gastric polyp'<br>#2. 'proton pump inhibitor'/exp OR 'proton pump inhibitor' OR 'inhibitors, proton pump' OR 'proton pump inhibitor' OR 'inhibitor, proton pump' OR 'inhibitor, proton pump' OR 'pump inhibitor, proton'<br>#3. #1 AND #2<br>#4. 'clinical':ti,ab AND 'trial':ti,ab OR 'clinical trial'/exp OR random* OR 'drug therapy':lnk<br>#5. #4 AND #3<br>Cochrane Central Register of Controlled Trials<br>((proton pump inhibitor OR Inhibitors, Proton Pump OR Proton Pump Inhibitor OR Inhibitor, Proton Pump OR Pump Inhibitor, Proton)) AND (((Stomach Neoplasms OR Neoplasm, Stomach OR Stomach Neoplasm OR Neoplasms, Stomach OR Gastric Neoplasms OR Gastric Neoplasm OR Neoplasm, Gastric OR Neoplasms, Gastric OR Cancer of Stomach OR Stomach Cancers OR Gastric Cancer OR Cancer, Gastric OR Cancers, Gastric OR Gastric Cancers OR Stomach Cancer OR Cancer, Stomach OR Cancers, Stomach OR Cancer of the Stomach OR Gastric Cancer, Familial Diffuse OR atrophic gastritis OR intestinal metaplasia OR Atrophic Gastritides OR Atrophic Gastritis OR Gastritides, Atrophic OR Enterochromaffin like Cells OR Enterochromaffin-like Cell OR ECL Cells OR ECL Cell OR gastric polyp))) Filters: Clinical Trial, Randomized Controlled Trial	Supplementary Table 1
Selection process	8	Two independent investigators screened titles and abstracts to include all potential studies in the search results. They independently screened them separately in full text to have eligible studies and document reasons for exclusion. When unsure of study inclusion, the two review authors discussed obtaining a solution, and if had been needed, a third expert was consulted.	95-98
Data collection process	9	Two independent investigators extracted data from a single study using a predesigned data extraction form. Extracted data included the first author's surname, year of publication, type of experiment, number of participants, age, sex, baseline diagnosis, inclusion and exclusion criteria, treatment and experimental interventions, time of intervention, main output, and number of events.	104-110
Data items	10a	Occurrence and progression of gastric cancer-related lesions (gastric cancer, atrophy, intestinal metaplasia, ECL cell hyperplasia, and gastric polyps)<br>Odds ratio (OR) between the occurrence and progression of each gastric cancer-related lesion and the use of PPI<br>All results that were compatible with each outcome domain in each study were sought. Data were collected from eligible studies that met the inclusion criteria. Excluded studies and studies without original data were not considered. The decision to collect results was based on the	84-91

Section and Topic	Item #	Checklist item	Location where item is reported
		inclusion criteria, which required studies to report data related to the occurrence and progression of gastric cancer-related lesions and provide sufficient data to estimate the odds ratio between each lesion and the use of PPI.	
	10b	For this study, in addition to the outcomes listed in Item 10a, data were also sought on the following variables:<br>Participant characteristics: Age, gender, baseline diagnosis, and inclusion/exclusion criteria.<br>Intervention characteristics: Type and dose of proton pump inhibitor, duration of intervention, and other treatment strategies used in the control group.<br>Study characteristics: Year of publication, type of experiment, number of participants, and funding sources.<br>Assumptions were made in cases where information on certain variables was missing or unclear. For example, if the dose of proton pump inhibitor was not explicitly stated, it was assumed to be the standard dose used in clinical practice. If information on funding sources was not reported, it was assumed that the study did not receive any funding. Any assumptions made were documented in the data extraction form.	104-110
Study risk of bias assessment	11	In this study, the Cochrane Risk of Bias tool was used to assess the risk of bias in the included studies. Two independent investigators assessed the quality of each study using the tool, and a third investigator resolved any differences. The tool was used to evaluate the risk of bias across six domains: selection bias, performance bias, detection bias, attrition bias, reporting bias, and other sources of bias.<br>The two reviewers worked independently to assess the risk of bias in each study, and any discrepancies were resolved through discussion or consultation with a third investigator if necessary. No automation tools were used in the process.<br>The methods used to assess risk of bias were in accordance with the PRISMA guidelines.	109-110, Figure2,3
Effect measures	12	For each outcome, the effect measure used in the synthesis or presentation of results was the odds ratio (OR) with a 95% confidence interval (CI) by using the stochastic effect model DerSimonian-Laird random effects. The outcome was the occurrence and progression of each gastric cancer-related lesion (gastric cancer, atrophy, intestinal metaplasia, ECL cell hyperplasia, and gastric polyps) associated with the use of proton pump inhibitors (PPIs).	114-116
Synthesis methods	13a	The inclusion criteria for this review were randomized controlled trials (RCTs) studying the long-term use of proton pump inhibitors (PPIs) in adult patients. The study required a group of participants on maintenance therapy with PPIs and a control group using other treatment strategies for more than six months of intervention. Participants in both groups underwent upper gastrointestinal endoscopy at the beginning and end of the trial. They reported the number or proportion of participants with gastric mucosal lesions such as gastric cancer, atrophy, intestinal metaplasia, ECL cell hyperplasia, and gastric polyps. The primary outcome was that the study could provide sufficient data to estimate the odds ratio (OR) between the occurrence and progression of each gastric cancer-related lesion and the use of PPI.<br>We excluded articles without original data, studies that did not report data related to "gastric cancer, atrophy, intestinal metaplasia, ECL cell hyperplasia, and gastric polyps", and studies with duplicate reports.<br>Two independent investigators screened titles and abstracts to include all potential studies in the search results. They independently screened them separately in full text to have eligible studies and document reasons for exclusion. When unsure of study inclusion, the two review authors discussed obtaining a solution, and if needed, a third expert was consulted.<br>Studies that met the inclusion criteria were grouped according to their study interventions and the outcome measures reported. We synthesized the data from studies that reported similar interventions and outcome measures, and only those studies that met the eligibility criteria were included in the synthesis.	84-98
	13b	In this systematic review, we extracted data from each eligible study using a pre-designed data extraction form, which included information such as study design, number of participants, age, sex, baseline diagnosis, treatment and intervention, time of intervention, main outcomes, and number of events. We assessed the risk of bias using the Cochrane risk of bias tool.<br>For the statistical analysis, we used Stata 16 software to perform the meta-analysis and generate forest plots. For the two-category variables, we assumed that patients who quit were negative, and we converted the data that only reported the change in the number of events and the possibility of repeated reporting to a positive outcome. We used the DerSimonian-Laird random-effects model to estimate the odds ratio (OR) with a 95% confidence interval (CI) for the binary data. We evaluated the overall sensitivity and specificity of the model to fully explain the	104-110<br>114-126

Section and Topic	Item #	Checklist item	Location where item is reported
		additional uncertainty related to inter-study differences in the effects of different interventions. We calculated the Q statistic, I^2, and P value to evaluate the heterogeneity of the effect sizes.<br>If the main results were highly heterogeneous, we planned to conduct a sensitivity analysis to assess the impact of individual studies on aggregate statistics by removing one study at a time from the meta-analysis. We also planned to conduct a subgroup analysis to evaluate the potential impact of the incident location, Helicobacter pylori infection, PPI dose, and exposure time, if possible.<br>If the number of included studies was sufficient ($\geqslant$10), we would explore possible publication bias by constructing a funnel plot of the effect size of each trial relative to the standard error, evaluating the asymmetry of the funnel plot using Begg and Egger tests, and defining significant publication bias as a p-value < 0.1.	
	13c	In our systematic review, we used forest plots to visually display the results of individual studies and syntheses. The forest plot displays the effect size estimate (odds ratio) and its corresponding confidence interval for each study included in the meta-analysis. The vertical line at the null value (OR = 1) indicates no difference between the intervention and control groups. The size of each square represents the weight of the study in the meta-analysis, and the diamond shape represents the overall effect size estimate and its confidence interval. We used Stata16 software to generate the forest plots. We also used tables to present the characteristics of each study and summary statistics, such as the number of participants, baseline characteristics, and outcome data. The tables included information about the study design, interventions, and quality assessment. We used descriptive statistics, such as means and standard deviations, to present continuous data, and frequencies and percentages to present categorical data.	112-126
	13d	If meta-analysis was performed, the results were synthesized using the random-effects model. The choice of the random-effects model was based on the assumption that the true effect size varies across studies due to differences in study design, patient population, and other factors. The method used to identify the presence and extent of statistical heterogeneity was the Cochrane's Q test, and the magnitude of heterogeneity was assessed using the I-squared (I^2) statistic. An I^2 value of 0% indicates no heterogeneity, while values of 25%, 50%, and 75% indicate low, moderate, and high heterogeneity, respectively.<br>The software package used for the meta-analysis wasStata16.	114-119
	13e	If the presence of statistical heterogeneity was detected, we planned to perform subgroup analysis and meta-regression analysis to explore possible causes of heterogeneity among study results. The subgroup analysis would have been performed based on prespecified factors such as age, sex, intervention characteristics, and study quality. Meta-regression would have been used to assess the effect of continuous variables, such as age or duration of intervention, on the magnitude of treatment effects. We would have used the software Stata16 to perform these analyses. If the data were deemed unsuitable for meta-analysis, we would have reported the results narratively	119-121
	13f	We conducted sensitivity analyses to assess the robustness of the synthesized results. Specifically, we performed a leave-one-out analysis by removing one study at a time and re-analyzing the data to determine if any individual study had a significant impact on the overall results. Additionally, we performed a subgroup analysis to evaluate the potential impact of incident location, Helicobacter pylori infection, PPI dose, and exposure time. We also evaluated the impact of different statistical models and inclusion/exclusion criteria on the results. Overall, these sensitivity analyses were critical in ensuring the validity and reliability of our findings.	121-123
Reporting bias assessment	14	In our study, we did not explicitly assess the risk of bias due to missing results in a synthesis arising from reporting biases. However, we planned to evaluate the potential publication bias by constructing a funnel plot of the effect size of each trial relative to the standard error and testing for asymmetry using Begg's and Egger's tests. We defined significant publication bias as a p-value less than 0.1. Additionally, we conducted sensitivity analyses to assess the impact of individual studies on the overall results. These methods may indirectly identify any potential bias due to missing results in the synthesis.	123-126
Certainty assessment	15	We employed the Cochrane risk of bias tool to evaluate the level of certainty (or confidence) in the body of evidence for each outcome. This approach takes into account factors such as risk of bias, inconsistency, imprecision, indirectness, and publication bias to determine the overall certainty of the evidence for each outcome.<br>To ensure accuracy, two independent investigators assessed the certainty of evidence for each outcome, and any disagreements were resolved through discussion with a third investigator. The certainty of evidence was rated as high, moderate, or low. A high certainty rating	109-110, Figure2,3

Section and Topic	Item #	Checklist item	Location where item is reported
		suggests that further research is unlikely to alter our confidence in the estimate of effect, while a low certainty rating indicates that further research is likely to have a significant impact on our confidence in the estimate of effect.<br>Using the Cochrane risk of bias tool, we created a summary of findings table and evaluated the overall certainty of the evidence for each outcome. The results were presented through a narrative synthesis supported by Cochrane risk of bias evidence profiles. Additionally, we provided an explanation for any downgrading of the certainty of evidence.	
**RESULTS**			
Study selection	16a	Initially, a total of 4868 studies were identified from electronic databases, out of which 802 were found to be duplicates. Following screening by title and abstract, a total of 85 studies were selected for further evaluation, finally, 10 studies remained.	129-131, figure1
	16b	8 reports could not be retrieved, and out of the remaining 77 studies, 62 were found to be non-randomized controlled trials (RCTs) or review articles. Additionally, 2 reports were found to be duplicates of the same study, while 3 studies were excluded due to incomplete data.	131-134, figure1
Study characteristics	17	Table 1 shows the characteristics of the included clinical trials.	Table1
Risk of bias in studies	18	Figure2,3 shows the assessments of risk of bias for each included study.	Figure2,3
Results of individual studies	19	See Figure4-10.	Figure4-8
Results of syntheses	20a	In the analysis regarding ECL cells, I^2=7.13%, and I^2=0 in the rest of the analyses. p>0.05 was seen in all Q-tests, and the minimum value in several studies was p=0.19. No significant heterogeneity was observed in our research.	258-259
	20b	ECL cell hyperplasia (OR 3.01; 95% CI 1.29 to 7.04; P value=0.01; I^2=7.13%; Q quality: P=0.37)<br>gastric mucosal atrophy (OR 1.01; 95% CI 0.55 to 1.85; P value=0.97; I^2=25.8%; Q quality: P=0.26)<br>intestinal metaplasia (OR 1.14; 95% CI 0.49 to 2.68; P value=0.76; I^2=35.21%; Q quality: P=0.19)<br>gastric polyps (OR 1.13; 95% CI 0.68 to 1.89; P value=0.64; I^2=0%; Q quality: P=0.67)<br>gastric cancer (OR 1.06; 95% CI 0.79 to 1.43; P value=0.71; I^2=0%; Q quality: P=0.45)	161-207
	20c	In the analyses of gastric atrophy and intestinal metaplasia, after including the data under extreme assumptions, I^2=25.8% for gastric atrophy and I^2=35.21% for intestinal metaplasia, and after excluding this data the I^2 decreased to 7.48% and 0, respectively, with the same direction of meta-analysis results before and after exclusion, suggesting that the scale of inclusion of data from this study does not affect the final conclusions of the analysis.	259-260
	20d	Atrophy: When re-analysed after excluding studies assuming extreme cases of Fiocca34, the results of the meta-analysis were in the same direction (OR 0.84; 95% CI 0.5 to 1.41; P value=0.51) and the I^2 decreased from 25.8% to 7.48%.<br>IM: After excluding studies that assumed extreme cases of Fiocca and conducting a subsequent analysis, the meta-analysis results were consistent in direction (OR 0.75; 95% CI 0.36 to 1.55; P value=0.44), and I^2 decreased from 35.21% to 0.	259-260
Reporting biases	21	See Figure2,3.	Figure2,3
Certainty of evidence	22	ECL cell hyperplasia (OR 3.01; 95% CI 1.29 to 7.04)<br>gastric mucosal atrophy (OR 1.01; 95% CI 0.55 to 1.85)<br>intestinal metaplasia (OR 1.14; 95% CI 0.49 to 2.68)<br>gastric polyps (OR 1.13; 95% CI 0.68 to 1.89)<br>gastric cancer (OR 1.06; 95% CI 0.79 to 1.43)	161-207

PRISMA 2020 Checklist

Section and Topic	Item #	Checklist item	Location where item is reported
**DISCUSSION**			
Discussion	23a	The main results of our analysis showed that the current study showed no evidence that long-term maintenance therapy with PPIs caused or exacerbated gastric mucosal atrophy, intestinal metaplasia, gastric polyps, and gastric cancer in users relative to other treatments but showed a correlation with ECL cell proliferation	210-213
	23b	Since the publication of relevant data differed among the literature, and some literature only published the prevalence rate per year, and we could not obtain the trend of its change. We did not receive a reply after sending an email to the authors to ask about it. We accumulated this data and included it in the analysis, due to no response to our email inquiries, which also resulted in some bias;<br>The intervention modalities of the control group formed in the study were diverse, including placebo, surgery, and other therapeutic drugs, the association with disease derived from the experimental group compared with different styles of the control group may not be stable as a consequence.	264-271
	23c	First, the number of literature included in this study is still relatively limited. We sought as much relevant literature as possible while ensuring the quality of the literature and included trials that had not yet been completed but for which data were published annually; second, the primary outcomes of some of the literatures did not match the present study. It mainly reported data of interest in the form of secondary results or adverse events found during the primary outcome, and we looked for reports of relevant events from the eligible full text, but there may still be incomplete or non-specific statistics for this part of the data; finally, some of the literature may also have factors of bias such as open-label design and pharmaceutical industry funding, as well as some aspects of bias that we have not yet identified.	261-276
	23d	Based on our meta-analysis of the available literature, we found no significant association between PPI maintenance therapy and an increased risk of gastric mucosal atrophy, intestinal metaplasia, gastric polyps, or gastric cancer. Therefore, our results suggest that PPI therapy can be used safely and effectively in managing patients with clinical gastrointestinal diseases who require long-term maintenance therapy. However, caution should still be exercised when considering PPI use, and the potential risks and benefits of treatment should be carefully evaluated for each individual patient.	281-287
**OTHER INFORMATION**			
Registration and protocol	24a	We registered at International Prospective Register of Systematic Reviews (number CRD42022379692)	99-101
	24b	The review protocol was registered with the International Prospective Register of Systematic Reviews (PROSPERO) database (registration number: CRD42022379692) and the protocol has been published [https://www.crd.york.ac.uk/prospero/display_record.php?ID=CRD42022379692].	99-101
	24c	We incurred no deviations from the a priori review protocol.	99-101
Support	25	Non-financial support for the review.	298
Competing interests	26	The authors declare that they have no competing interests.	289-290
Availability of data, code and other materials	27	All template data collection forms, data extracted from the included studies, data used for all analyses, analytic code, and any other materials used in the review can be obtained by contacting the authors in a reasonable manner.	

From: Page MJ, McKenzie JE, Bossuyt PM, Boutron I, Hoffmann TC, Mulrow CD, et al. The PRISMA 2020 statement: an updated guideline for reporting systematic reviews. BMJ 2021;372:n71. doi: 10.1136/bmj.n71
For more information, visit: http://www.prisma-statement.org/
